# Supplementary material for: Immunogenicity of COVID-19 Vaccinations in Hematological Patients: 6-Month Follow-Up and Evaluation of a 3rd Vaccination
Source: Cancers (Basel). 2022 Apr 13;14(8):1962. doi: 10.3390/cancers14081962 (PMC9032993; doi:10.3390/cancers14081962)
Supplement: Supplementary file 1 [file cancers-14-01962-s001.zip › cancers-1660863-supplementary.pdf]

Supplemental Data

Immunogenicity of Covid-19 vaccinations in hematological patients: 6-month follow-up and evaluation of a 3rd vaccination

Supplemental Table

Supplemental Table S1. Multivariate analysis, adjusted for age and sex, describing the association of the risk factors with serological and cellular response.

Multivariate logistic regression for seroconversion (adjusted for age and sex)				
	Seroconversion (Anti-SARS-CoV-2 S ≥ 0.8)		T-cell response (≥ 45.95 SFCs/10^6 PBMC)	
	OR (CI)	p-value	OR (CI)	p-value
CD8+ <280/µl	0.76 (0.24 - 2.36)	0.629	2.56 (0.43 - 22.42)	0.332
CD4+ <310/µl	0.05 (0 - 0.32)	0.008	0.33 (0.04 - 2.52)	0.283
CD19+ <110/µl	0.13 (0.04 - 0.4)	0.000	1.86 (0.34 - 10.45)	0.469
Anti-CD20 therapy	0.08 (0.02 - 0.28)	0.000	1.23 (0.21 - 7.75)	0.821
Other therapy	1.98 (0.46 - 10.44)	0.376	0.71 (0.08 - 7.2)	0.752

Supplemental Figure

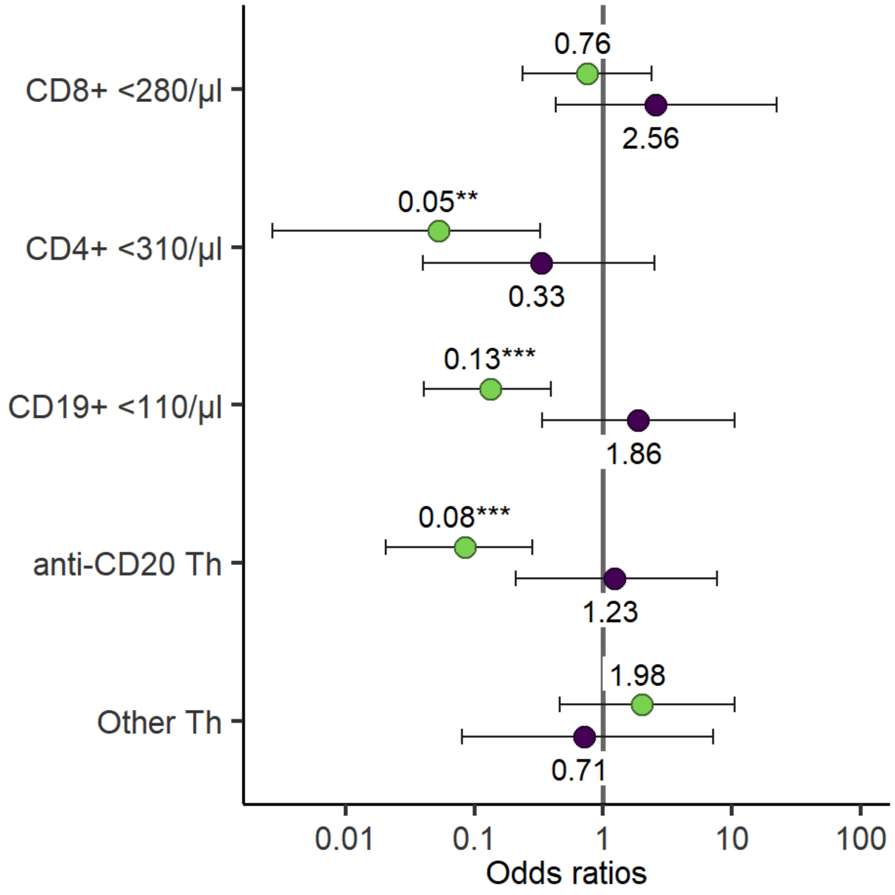

Supplemental Figure S1. Odds ratio for multivariate logistic regression, adjusted for age and sex, assessing seroconversion and T-cell response. Values for seroconversion are highlighted by the green bars. Values for T-cell response are highlighted by the purple bars.
